# Clinical Signs and Symptoms of Anxiety Due to Adverse Childhood Experiences: A Cross-Sectional Trial in Adolescents

**DOI:** 10.3390/healthcare12151515

**Published:** 2024-07-30

**Authors:** Aikaterini Marini, Ignatia Farmakopoulou, Ioannis Dritsas, Evgenia Gkintoni

**Affiliations:** 1 Department of Medicine, University of Athens, 11527 Athens, Greece; katerinamarini@hotmail.com; 2 Department of Education and Social Work, University of Patras, 26504 Patras, Greece; ifarmakop@upatras.gr (I.F.); dritsasioan@upatras.gr (I.D.); 3 Department of Psychiatry, University General Hospital of Patras, 26504 Patras, Greece

**Keywords:** adverse childhood experiences, clinical psychology, psychopathology, anxiety, psychological assessment, adolescence, COVID-19

## Abstract

Background: Adverse Childhood Experiences (ACEs) are linked to the development of anxiety in adolescence. According to recent studies, the COVID-19 pandemic represents a novel ACE that is associated with anxiety among adolescents. This study investigates the relationship between ACEs, COVID-19, and anxiety in adolescents. Methods: A cross-sectional study was conducted using a community sample of 248 boys and girls ages 12 to 15 years (mean = 13.50 years) from five high schools in Eastern Attica. A total of four questionnaires were used: (1) Demographic Questionnaire, (2) State–Trait Anxiety Inventory for Children—STAIC, (3) Adverse Childhood Experiences Scale, and (4) COVID-19 Impact Scale. Results: The results demonstrated a moderate association between the total number of ACEs and anxiety (trait and state) in adolescence (trait anxiety: *ρ* = 0.37, *p* < 0.001; state anxiety: *ρ* = 0.29, *p* < 0.001). Girls scored significantly higher than boys on both trait anxiety (U = 4353, *p* < 0.001; mean difference = 5.5) and state anxiety (U = 5822.5, *p* = 0.014; mean difference = 2). The number of ACEs was found to be significantly related to the impact of COVID-19 *(β* = 0.025, *p* < 0.001). Conclusions: This study highlights the significant link between ACEs and increased anxiety in adolescents, which is further exacerbated by the COVID-19 pandemic. The findings indicate that girls are more affected than boys. These results emphasize the need for targeted mental health interventions to enhance coping mechanisms, reduce stress, and address anxiety in adolescents, particularly during global crises like the pandemic. Developing such programs is essential for supporting the mental well-being of youth facing multiple stressors.

## 1. Introduction

Adverse Childhood Experiences (ACEs) are defined as events that occur between birth and the end of adolescence (0–18 years of age) and which have the potential to cause psychological trauma. ACEs include abuse, neglect, and family dysfunction [1] and are influenced by various cultural, social, and environmental factors that impact the mental and physical health of young people around the world [2]. The conceptual framework of ACEs has been expanded to include exploitation, poverty, loss, terrorism, natural disasters, and discrimination.

Although studies on the consequences of ACEs in childhood and adolescence are limited [3], they nevertheless provide valuable insight detailing the relationship of ACEs with mental and physical disorders, such as the comorbidity of depressive and anxiety disorders, suicidality, substance abuse, and obesity [4,5,6]. Additionally, other researchers [7] have suggested that ACEs could cause permanent damage to the developing brain and alter the function of the immune and neuroendocrine systems, putting young people at high risk for developing chronic physical and mental disorders [8]. ACEs are, therefore, an essential risk factor for the physical and mental health of children and adolescents [9].

Anxiety is one of the most frequently diagnosed mental disorders in children and adolescents, with a course that is a common trait into adulthood [10]. In the United States, the National Survey of Children’s Health (NSCH, 2016), 7.1% of children and adolescents ages 3 to 17 years reported anxiety symptoms [11]. In another US-based survey, the National Comorbidity Study–Adolescent Supplement (NCS-A, 2011), the lifetime prevalence of anxiety disorders in adolescents between the ages of 13 and 18 years was 31.9% [12].

A substantial portion of the research examining the association between ACE and the occurrence of anxiety symptoms in adolescents is based on parent/guardian reports. However, there is a growing tendency to also gather information from the focal child or adolescent. ACEs are also studied in terms of their total number and their classification. Commonly studied ACEs include parental divorce/separation, losing a parent, having a parent with cancer, having a family member with a mental illness, family substance use, witnessing domestic violence, exposure to community violence, maltreatment due to racism, and economic hardship [13,14,15,16]. In general, ACEs are categorized into four groups: divorce/parental separation, family dysfunction, exposure to violence, and financial hardship [4,6,17].

Epidemiological studies continue to highlight a relationship between ACEs and anxiety in adolescents. For instance, a large-scale study conducted in China with adolescents found that ACEs were positively associated with anxiety symptoms [18]. In this study, 23.41% of the adolescents reported peer rejection, 20.92% emotional abuse by a parent, and 17.86% emotional neglect by family. In a separate study using the US NSCH, 9% of adolescents diagnosed with current anxiety had experienced four or more ACEs, with parental divorce/separation being the most prevalent ACE [4]. Many researchers [6,19] have reported similar findings in large-scale epidemiological studies of adolescent populations with a previous or current diagnosis of anxiety or depressive disorder.

In addition, recent literature has suggested that the COVID-19 pandemic disrupted development in adolescents. This disruption was associated with several negative psychosocial and psychological outcomes, including the emergence of anxious symptomatology [20,21,22]. A systematic review of 21 studies (*n* = 33,398) on the psychosocial consequences of COVID-19 in children, adolescents, and young adults demonstrated that excess worrying, irritability, home confinement, and fear of COVID-19 infection and transmission were associated with mild to severe anxiety symptoms during the COVID-19 pandemic [23]. Moreover, a meta-analysis pooling 29 studies (mean age of participants was 13) reported the pooled prevalence of clinically elevated depression and anxiety symptoms was 25.2% and 20.5%, respectively [24]. If these findings are compared to pre-pandemic estimates, it suggests that youth mental health difficulties, such as anxiety and depression, may have doubled during the COVID-19 period. This significant increase highlights the profound impact the pandemic has had on the mental health of children and adolescents, exacerbating pre-existing conditions and creating new challenges for mental health treatment providers. One other study [22] concluded that adolescents could experience both short- and long-term negative consequences associated with COVID-19. Thus, it is of paramount importance that we learn more about whether COVID-19 was perceived as stressful to youth and, furthermore, whether COVID-19 was associated with higher than usual levels of anxiety. Assessing the impact of COVID-19 on the mental health of adolescents can help mental health providers address the specific needs of vulnerable populations [25,26,27,28].

### Aims of the Present Study

Given that ACEs may probably occur in the lives of individuals from the beginning of their development [23] and that anxiety is one of the most diagnosed mental disorders in adolescents [10], the primary purpose of this study was to examine the relationship between ACEs and anxiety symptoms in a sample of Greek adolescents. Based on the literature, we hypothesized that ACEs would be positively associated with anxiety in adolescence. Having considered the fact that the coronavirus COVID-19 pandemic has been recently recognized as a new ACE [24], a second goal of the current study was to examine the relationship between the impact of the COVID-19 pandemic on adolescents as well as the relationship between the number of ACEs and the impact of the COVID-19 pandemic.

Therefore, we hypothesized that the number of ACEs would be related to the impact of the COVID-19 pandemic and that the impact of the COVID-19 pandemic would be related to adolescent anxiety [4,6,13]. Numerous studies have examined the relationship between demographic factors and anxiety in adolescents. Research consistently indicates that females report higher levels of anxiety than males [4]. Age may also be significantly associated with both ACEs and anxiety. For instance, one study showed that anxiety disorders emerge around 14 years of age [12]. Based on the literature, we also hypothesized that female adolescents would report more anxiety than male adolescents [29]. Collectively, these findings underscore the importance of considering demographic factors in understanding and addressing anxiety in adolescents. In the following section, the description of the research hypotheses is presented as follows:

- [RH1] The total number of ACEs will be positively associated with anxiety symptoms in adolescents.
- [RH2] The number of ACEs will be positively associated with the perceived negative impact of the COVID-19 pandemic.
- [RH3] The perceived negative impact of the COVID-19 pandemic will be positively associated with anxiety symptoms in adolescents.
- [RH4] Anxiety among the sample of Greek adolescents will be related to the following three demographic factors: age, gender, and family income.
- [RH5] The perceived negative impact of the COVID-19 pandemic on adolescents will be related to the following three demographic factors: age, gender, and family income.
- [RH6] Adolescent girls will report higher levels of anxiety than adolescent boys.

## 2. Materials and Methods

This study employed a cross-sectional design to examine the relationship among Adverse Childhood Experiences (ACEs), the COVID-19 pandemic, and anxiety symptoms in adolescents. A community sample of N = 248 boys and girls aged 12 to 15 years (mean = 13.50 years) from five high schools in Eastern Attica was selected using stratified random sampling to ensure a representative distribution of key demographic variables such as age and gender. Data were collected through four structured questionnaires: a Demographic Questionnaire, the State–Trait Anxiety Inventory for Children (STAIC), the Adverse Childhood Experiences Scale, and the COVID-19 Impact Scale.

Additionally, the study employed stratified random sampling. Stratified random sampling was chosen to enhance the representativeness of the sample and to ensure that key demographic variables, such as age and gender, were proportionately represented. This method allows for more precise estimates of the population parameters and enhances the generalizability of the study findings.

By detailing the stratification variables and the random sampling process, we ensured that the sample was diverse and representative of the adolescent population in Eastern Attica. This methodological rigor supports the validity and reliability of our study outcomes, providing a robust basis for our conclusions regarding the relationship between adverse childhood experiences, COVID-19 impact, and anxiety in adolescents.

The principals of the Mainstream High schools sent out an electronic document to the parents/caregivers of the students. This notification was intended to facilitate research and provide relevant study information. Additionally, a consent form was given to all parents of children attending five schools that chose to participate in the study. Following receipt of consent from parents, a schedule was established to administer the survey questionnaires to students. To conduct the current cross-sectional study, approval was obtained from the Ethics Committee of the University of Athens, Faculty of Medicine, Greek Academy of Sciences. Following ethics review approval, additional approval to participate was obtained from five principals of High Mainstream Schools located in the Municipality of Eastern Attica, who agreed to take part in the study.

### 2.1. Measures

#### 2.1.1. Self-Designed Demographic Information Questionnaire

A total of eight demographic questions assessed the students’ gender, age, ethnicity, education year, infection by COVID-19 and its frequency, whether parent(s) tested positive for COVID-19 and how many times, and family income filled out by adolescents.

#### 2.1.2. Adverse Childhood Experiences Questionnaire

The Adverse Childhood Experiences Questionnaire was derived from the K-SADS-PL [30,31], which is translated and adapted to the Greek language with the written permission of the instrument’s authors. The instrument examines the following serious ACEs: car or other accident, witnessing a fire or other natural disaster, witnessing or being a victim of a violent crime, receiving traumatic news, witnessing domestic violence, and being a victim of physical or gender-based abuse. The questionnaire is comprised of 11 questions with Yes–No response options. Questions 10 and 11, which attend to gender-based violence victimization, were eliminated in light of ethical and moral considerations because they belong to sensitive data categories.

#### 2.1.3. State–Trait Anxiety Inventory for Children (STAIC)

The State–Trait Anxiety Inventory for Children (STAIC) [32] consists of a state anxiety subscale (A-State) and an anxiety predisposition subscale (A-Trait). Permission was granted from the original authors to use a Greek-translated version of the instrument. The Anxiety Predisposition Scale (A-Trait) consists of 20 statements indicating the presence of anxiety symptoms, to which children respond on a three-point Likert-type scale: “Very often”, “Sometimes”, and “Rarely” for the frequency of occurrence of the behavior described in each sentence. Permission was granted from the original authors to use a Greek-translated version of the instrument.

In the Greek standardized version [33], the internal consistency (Cronbach alpha) and test–retest reliability indices of the scale among the Greek population are α = 0.80 and α = 0.81, respectively. In this study, Cronbach’s alpha reliability coefficient for both subscales was α = 0.88, indicating adequate reliability. Specifically, the A-Trait scale’s Cronbach’s alpha was α = 0.85, and the A-State scale’s Cronbach’s alpha was α = 0.86, both above the acceptable limit of 0.70 [34]. By focusing on the two dimensions of anxiety—trait and state—the STAIC provides a comprehensive assessment of both the frequency and intensity of anxiety symptoms.

#### 2.1.4. Questionnaire on the Impact of COVID-19 Pandemic

For the purpose of this study, the authors developed a self-report questionnaire with 13 questions to assess the negative experiential, cognitive-learning, and affective effects of the COVID-19 pandemic on the lives of adolescents. The questions are answered on a three-point Likert-type scale, “Not at all”, “A little”, “A lot”, and scored 0, 1, 2, respectively. The students were questioned about their parents’ employment, their mental state during the pandemic, and any alterations to their daily routine. They were also asked about changes in the quality of their relationships with their parent(s) and their sibling(s) and their school performance during the pandemic. The COVID-19 Impact Scale assesses three facets of how the pandemic influences adolescents’ functioning: psychosocial, educational, and economic. For example, a question from the psychosocial facet might ask, ‘How often did you feel anxious or stressed due to the pandemic?’ An example from the educational facet could be, ‘Did the pandemic affect your ability to complete school assignments?’ Finally, a question from the economic facet might include, ‘Did the pandemic cause any financial difficulties for your family?’ These questions demonstrate how COVID-19 has influenced adolescents in multiple aspects of their lives [35]. They were also asked about changes in the quality of their relationships with their parent(s) and their sibling(s) and their school performance during the pandemic.

A principal component analysis (PCA) with varimax rotation was performed on the COVID-19 Impact Scale. This analysis was conducted to explore the underlying dimensions of the scale, hypothesizing that different aspects of COVID-19′s impact on adolescents’ lives could be captured by separate factors. The PCA revealed a three-factor solution, explaining a total of 62% of the variance. The Kaiser–Meyer–Olkin measure verified the sampling adequacy for the analysis, *KMO* = 0.85, and all KMO values for individual items were above 0.6, which is above the acceptable limit of 0.5. Bartlett’s test of sphericity was significant *χ*^2^(78) = 834.25, *p* < 0.001, indicating that correlations between items were sufficiently large for PCA.

### 2.2. Analyses

In this study, the Statistical Package for the Social Sciences (SPSS 22.0) was utilized for data processing and analyses. We assessed the normality of quantitative (continuous) variables using the Kolmogorov–Smirnov test. Normally distributed variables were described using mean values and standard deviations (SD), while non-normally distributed variables were characterized by medians and interquartile ranges. Absolute (N) and relative (%) frequencies were used to describe categorical variables. We used the Mann–Whitney test to compare differences in the distributional properties of quantitative variables between two groups. These non-parametric tests are particularly suited for analyzing ordinal data, like Likert scale responses, as they evaluate ranks and thus provide insights into group medians, which are useful when data contain outliers that might skew means. Additionally, logarithmic transformations were applied to skewed data to linearize relationships and stabilize variance across different groups or factor levels. This approach facilitates analysis with many statistical methods and ensures robustness, especially given the non-normal distribution of specific data points.

We applied the Bonferroni correction to mitigate the risk of Type I errors resulting from multiple comparisons. We set the significance level as the *p*-value (0.05) divided by the number of comparisons (k). To examine relationships between two continuous variables, Spearman’s correlation coefficient (ρ) was utilized. Comparisons of categorical variables between male and female students were conducted using Pearson’s chi-square tests; however, comparisons did not include individuals identifying as ‘Other’ due to their small sample size. Multivariate linear regression analyses were conducted to identify factors associated with the Negative Effects of the COVID-19 pandemic scale and the anxiety dimensions of the STAIC questionnaire. These analyses included demographic and family data, the number of Adverse Childhood Experiences (ACEs), and the impact of COVID-19 as independent variables. The dependent variables were the Negative Effects scale and the STAIC anxiety dimensions, respectively. Additionally, a stepwise method was used where variables with a significance level of 0.05 or less were included in the model, and those with a significance level above 0.10 were excluded. This method ensures that only statistically significant predictors are retained in the final models, facilitating a clearer interpretation of the results.

## 3. Results

The study questionnaires were distributed to 315 high school students, 12–15 years old (male, female, and other) from the Secondary Education Directorate of Eastern Attica. Two hundred forty-eight (N = 248) students (representing 78.7% participation) returned completed questionnaires. Students completed these questionnaires during a designated extracurricular period. The estimated duration for completion was between 15 and 20 min. The mean age of the sample was 13.50 years of age (standard deviation = 1.0). More than half of the sample was female, 54.8%, and 2.8% identified as non-binary. Due to their small sample size, “Other sex identity” participants were excluded from statistical analyses involving the “gender” variable.

Based on the analysis, the results show that 87.5% of the students were Greek, 84.7% had siblings, 85.1% lived with both biological parents, and 85.5% of the students were of middle economic level (annual family income approximately 20,000–40,000 EUR). In addition, 69% of students had their parents working, and 45.6% had a family member who had contracted COVID-19 (Table 1).

The total number of ACEs included in this study was nine. The types and percentages of ACEs experienced by the adolescents are listed as follows: (1) Flood/Earthquake/Shipwreck/Other Disaster experience (69.8%), (2) Experience of Unexpected News of Illness/Death of a Loved One (69.4%), (3) Experience of Robbery/Exposure to Gunshot/Shoplifting/Assault on a Third Party (30.6%), (4) Experience of Personal Assault by a Third Party with Risk of Serious Injury (19.8%), (5) Domestic Violence between Parents (18.1%), (6) Domestic Violence of Parents against the Child (10.1%), (7) Self-Injury in Other Accident (9.7%), (8) Injury to Self/Other in a Car Accident (8.9%), (9) Fire Experience (8.1%). No significant differences were found between males and females in terms of the number of ACEs, *p* > 0.05.

Notably, most students had experienced at least two ACEs, as 69.4% had an unexpected experience, such as illness/death of a loved one, and 69.8% had experienced a flood/earthquake/wreck or other disaster. Furthermore, 18.5% of the students had a family member facing a health problem. COVID-19 affected every aspect of students’ lives, including adverse consequences that resulted from distance learning. The greatest effects of COVID-19 on students’ lives included distance learning (66.9%), screen time during COVID-19 (67.3%), and no change in the quality of family relationships (56%). Sixty-two percent of students (62%) reported that COVID-19 had little or no effect on family tension/conflict. Also, 62.9% of the students reported no effect of COVID-19 on the frequency of waking up during the night. The Impact of the COVID-19 pandemic score (Table 2) ranged from 0.24 to 1.59 points, with a mean value of 0.91 points (SD = 0.27 points).

The impact of the COVID-19 pandemic score by gender is given in Table 3. Girls reported significantly more negative effects from the pandemic than boys, U = 4062.5, *p* < 0.001.

Table 4 below gives the students’ scores for the two dimensions of the STAI-C Anxiety scale, where higher values indicate more anxiety.

The levels of trait and state anxiety are given in Table 5 below. As previously mentioned, three cut points were used to classify students on the basis of their reported anxiety symptoms.

Table 6 below gives the students’ scores on the dimensions of the STAI-C Anxiety scale by gender. Girls reported significantly more trait anxiety (U = 4353, *p* < 0.001) and state anxiety (U = 5822.5, *p* = 0.014).

Table 7 shows the Spearman correlation coefficients for the two anxiety dimensions with age, the number of ACEs, and the scale of Negative Effects from the COVID-19 pandemic.

The two dimensions of anxiety were positively related to the number of ACEs, and likewise, both dimensions of anxiety were positively related to the scale measuring the negative effects of COVID-19.

Table 8 below shows the scores of the students in the trait anxiety dimension based on demographics and family situational measures, including parental education level.

Students of low/middle economic level (U = 2435, *p* = 0.023) and those with a member in their family with a health problem (U = 3613.5, *p* = 0.019) had significantly more trait anxiety. Also, the trait anxiety score differed significantly in relation to the number of rooms in the student’s home, χ^2^(2) = 10.17, *p* = 0.006. Students who lived in smaller houses reported significantly more trait anxiety, and this finding held up with a Bonferroni correction (*p* = 0.002).

At this point, it is important to mention that the score of the students in the state anxiety dimension concerning their demographic and family information was similar to that of trait anxiety and, for this reason, is not depicted in this paper. More specifically, students of low/middle economic level reported significantly more state anxiety (U = 2545, *p* = 0.049), as well as those who had a family member facing a health problem (U = 3652, *p* = 0.023). Also, the state anxiety score differed significantly depending on the number of rooms student’s home had, the χ^2^(2) = 7.65, *p* = 0.022 and class, the χ^2^(2) = 8.66, *p* = 0.013. Specifically, after the Bonferroni correction, students who lived in two-bedroom houses reported significantly more state anxiety compared to students who lived in four-bedroom houses or other number-bedroom houses (*p* = 0.008). Also, first-year high school students reported significantly less state anxiety than second-year high school students (*p* = 0.004).

Table 9 contains the results of the multivariate linear regression modeling the negative effects of the COVID-19 pandemic as the dependent variable.

Gender, with females as the reference category, was significantly associated with the negative effects of COVID-19. Gender and multiple ACEs accounted for 21.0% of the variance in the NE of the COVID-19 pandemic scale. Table 10 and Table 11, respectively, contain the results of the multivariate linear regression with the dimensions of trait and state anxiety as dependent variables.

The variables in Table 10 explained a significant portion of the variance in trait anxiety. Specifically, gender, the number of ACEs, and the scale of Negative Effects from the COVID-19 pandemic were found to be independently and significantly related to the dimension of trait anxiety.

The variables in Table 11 explained a significant portion of the variance in state anxiety. Specifically, grades in school, the number of ACEs, and the scale of Negative Effects from the COVID-19 pandemic were found to be independently and significantly related to the dimension of state anxiety.

## 4. Discussion

The findings of this study confirm RH1 in that the total number of ACEs is positively associated with anxiety symptoms in adolescents [4,36,37,38,39,40,41]. There was a clear positive correlation between the total number of ACEs and both trait and state anxiety in adolescents [RH1]. In addition, in support of RH2, we found that the number of ACEs is positively associated with the perceived negative impact of the COVID-19 pandemic [5,42]. The findings support this hypothesis, showing that the number of ACEs significantly relates to the perceived negative impact of the pandemic [RH2].

The perceived negative impact of the COVID-19 pandemic is also positively associated with anxiety symptoms in adolescents [24,43]. There was a strong correlation between the negative impacts of COVID-19 and increased levels of trait and state anxiety [RH3] [44,45,46]. At this point, it is important to consider that adolescents who already had trait and state anxiety with the emergence of a further stressor—such as, in this case, COVID-19—were expectedly influenced by the perceived negative impact of the pandemic.

It has been found that anxiety among Greek adolescents is related to the following three demographic factors: age, gender, and family income. Certain demographic factors are clearly related to anxiety levels [47,48,49]. For example, students of low/middle economic level and those with a family member facing a health problem had significantly more trait anxiety [50]. Additionally, anxiety levels differed significantly depending on the number of rooms in the student’s home and the class level [RH4]. It is important to try to put oneself in the place of the teenagers who lived in small apartments during the difficult period of quarantine. Let us recall that adolescents could not follow their daily routine, i.e., going to school and/or their extracurricular activities, such as participation in a sport. In practical terms, this meant that they necessarily had to share a room with their younger or older siblings and, therefore, were unable to have even a little personal space and time to themselves. This fact, in itself, can increase anxiety levels in the already overburdened psychological state of a number of participants in this study.

The perceived negative impact of the COVID-19 pandemic on adolescents is also related to these demographic factors. The study shows that the negative effects of COVID-19 were more significantly reported by female students compared to male students. Gender and multiple ACEs accounted for a significant part of the variance in the Negative Effects of COVID-19 pandemic scale [RH5].

Adolescent girls report higher levels of anxiety than adolescent boys. This hypothesis is confirmed by the study, which shows that girls reported significantly higher levels of both trait and state anxiety compared to boys [RH6].

These associations indicate that the study’s findings align well with the proposed research hypotheses, providing robust evidence for the relationships among ACEs, the impact of the COVID-19 pandemic, demographic factors, and anxiety symptoms in Greek adolescents.

Adverse Childhood Experiences (ACEs) are critical factors influencing the psychological well-being of adolescents. ACEs, such as abuse, neglect, and household dysfunction, are known to have long-term effects on mental health, including the development of anxiety disorders. This study investigates the relationship between ACEs, the impact of the COVID-19 pandemic, and anxiety symptoms in adolescents from Eastern Attica, Greece.

The study found a significant positive correlation between the total number of ACEs and both trait and state anxiety in adolescents. This aligns with previous research indicating that higher exposure to ACEs increases the risk of developing anxiety symptoms [4].

The COVID-19 pandemic has been identified as a novel ACE, significantly affecting adolescents’ mental health. The study revealed that the perceived negative impact of COVID-19 is positively associated with higher levels of anxiety. Adolescents who experienced multiple ACEs perceived the pandemic more negatively, exacerbating their anxiety symptoms [24].

Female adolescents reported significantly higher levels of both trait and state anxiety compared to their male counterparts. This finding is consistent with broader literature suggesting that females are more prone to anxiety disorders due to various socialization patterns and coping mechanisms [49].

Adolescents from lower socioeconomic backgrounds reported higher levels of anxiety. Financial instability and related stressors during the pandemic likely contributed to these elevated anxiety levels, underscoring the importance of socioeconomic status in mental health outcomes [41].

The study highlighted that adolescents who lacked effective coping mechanisms or had adverse family environments (e.g., illness of a family member) were more vulnerable to increased anxiety. This finding emphasizes the need for supportive family dynamics and effective coping strategies to mitigate the adverse effects of ACEs and pandemic-related stress [39]. Early intervention and supportive family dynamics are essential in enhancing outcomes for adolescents facing mental health challenges [51].

The findings of this study underscore the profound impact of ACEs and the COVID-19 pandemic on adolescent mental health. The strong associations among the number of ACEs, the negative impact of the pandemic, and elevated anxiety levels highlight the urgent need for targeted mental health interventions. These interventions should focus on building resilience, providing psychological support, and addressing socioeconomic disparities to improve the mental health outcomes of adolescents.

There are a few limitations to this study worth noting. The small sample size limits the statistical power, particularly for conducting gender-specific analyses, and could potentially reduce the generalizability of the findings. Although reliable, the use of a non-validated COVID-19 impact measure restricts the conclusions that can be drawn from the results. Future studies should employ larger samples and validated instruments, such as the COVID-19 Anxiety scale [19], to enhance the validity and reliability of the findings.

In conclusion, this study provides valuable insights into the interplay between ACEs, the perceived impact of the COVID-19 pandemic, and anxiety in Greek adolescents. The findings emphasize the cumulative nature of stressors and the significant role of socioeconomic and gender factors in shaping mental health outcomes. These results underscore the urgent need for comprehensive, validated research and targeted interventions to mitigate the adverse effects of ACEs and pandemic-related stress on adolescent mental health [52,53,54,55]. Future research should address the current study’s limitations by incorporating more extensive, representative samples and robust measurement tools, thus providing a more substantial evidence base for effective intervention strategies [56,57,58,59,60].

### Future Research Recommendations

Future research should consider longitudinal and prospective designs to better understand the long-term effects of the COVID-19 pandemic and ACEs on adolescent anxiety. Such studies could identify potential protective and risk factors and other long-term consequences on mental and physical health. Furthermore, validating the self-report scale in different adolescent samples could improve its reliability and generalizability. Exploring ACEs by type rather than solely by number and comparing groups with and without diagnosed anxiety disorders would offer valuable information for targeted interventions and prevention strategies in adolescent populations. Additionally, examining variables with groupings of ACEs according to their type (e.g., interpersonal ACEs, sociodemographic ACEs) could yield helpful information for diagnosis, prevention, and intervention. Considering gender as a risk factor for anxiety is crucial, as female adolescents are emerging internationally as a high-risk group for developing anxiety symptoms. This approach aligns with the need for comprehensive, validated research and targeted interventions to mitigate the adverse effects of ACEs and pandemic-related stress on adolescent mental health.

## 5. Conclusions

In conclusion, this study underscores the significant relationships between Adverse Childhood Experiences (ACEs), the COVID-19 pandemic, and anxiety levels in adolescents, particularly emphasizing the heightened vulnerability of female adolescents and those from lower socioeconomic backgrounds. The cumulative nature of these stressors necessitates targeted interventions that enhance coping mechanisms, mitigate stress, and address the mental health challenges induced by both ACEs and pandemic-related stressors. The findings advocate for the implementation of universal prevention programs and stress the importance of supportive family dynamics in promoting adolescents’ mental well-being. Future research with larger samples and validated tools is essential to refine these interventions and provide robust support systems for adolescents grappling with anxiety and stress. This comprehensive approach will be pivotal in fostering resilient mental health outcomes among adolescents in the face of ongoing and future adversities.

## Figures and Tables

**Table 1:** Sample characteristics.

Characteristic	N (%)
Total Participants	248 (100)
Age (Mean ± SD)	13.50 ± 1.0
Gender—Male	112 (45.2)
Gender—Female	136 (54.8)
Gender—Non-binary	0 (0)
Ethnicity—Greek	217 (87.5)
Ethnicity—Other	31 (12.5)
Living with both biological parents	211 (85.1)
Siblings—Yes	210 (84.7)
Siblings—No	38 (15.3)
Socioeconomic Status—Low/Medium (20,000–40,000 EUR/year)	212 (85.5)
Socioeconomic Status—High	36 (14.5)
Parents’ Employment—Both Working	171 (69.0)
Family Member Contracted COVID-19	113 (45.6)

**Table 2:** Rating description of negative effects of the COVID-19 pandemic.

	Min	Max	Mean (SD)	Median
Scale of negative effects of COVID-19 pandemic	0.24	1.59	0.91 (0.27)	0.90 (0.69–1.10)

**Table 3:** Description of rating of negative effects of COVID-19 pandemic by gender.

	Sex		
		Female	Male
		Median	Median
Rating of impacts from the COVID-19 pandemic		1.00 (0.76–1.21)	0.79 (0.62–0.93)

**Table 4:** Student ratings on dimensions of the STAIC Anxiety scale.

	Min	Max	Mean (SD)	Median
Trait Anxiety	20.0	57.0	36.3 (7.7)	36 (31–42)
State Anxiety	20.0	55.0	32.3 (6.5)	31 (28–36)

**Table 5:** Trait and state anxiety levels.

		Ν	%
Trait Anxiety levels			
	Low	88	35.5
	Moderate	126	50.8
	High	34	13.7
State Anxiety levels			
	Low	150	60.5
	Moderate	87	35.1
	High	11	4.4

**Table 6:** Student scores on the two dimensions of STAIC Anxiety scale by gender.

	**Sex**		
	**Males**	**Females**	
	**Median**	**Median**	**U**
Trait Anxiety	33 (29–37)	38.5 (32.5–45)	4353 ***
State Anxiety	30 (28–33)	32 (28–37)	5822.5 *

* *p* < 0.05, *** *p* < 0.001.

**Table 7:** Spearman’s correlation coefficients of anxiety dimensions.

	Trait Anxiety	State Anxiety
Age	0.01	0.10
ACEs	0.37 ***	0.29 ***
Negative effects of COVID-19	0.54 ***	0.35 ***

*** p < 0.001.

**Table 8:** Student scores on the dimension of trait anxiety.

		Trait Anxiety	U Value
		Median	
Class	1st middle school	35 (30–41)	1.87 +
	2nd middle school	36.5 (31–44)	
	3rd middle school	35 (31–40)	
Siblings	No	38 (31–43)	3589
	Yes	35 (31–42)	
Living with both biological parents	No	39 (32–43)	3224.5
	Yes	35 (31–42)	
Financial level	Low/Medium High	36 (31–43)	2435 *
		33.5 (29–37)	
Father’s educational level	Elementary/middle school/high school	36 (31–43)	6206
	University	35 (30–40)	
Mother’s educational level	Elementary/middle school/high school	35 (31–42)	7247
	University	36.5 (30–43)	
Both parents work	No	37 (31–42)	6188
	Yes	36 (30–42)	
The house where I live with my family is	1-bedroom house	38 (33–45)	10.17 +**
	2-bedroom house	36 (30.5–44)	
	3-bedroom house/Other	34 (30–38)	
A member of your family is facing a health problem	No	35 (30–41)	3613.5 *
	Yes	39 (32–45)	
Has any member of your family been sick with COVID-19	No	36 (31–42)	7139.5
	Yes	34 (31–42)	

+ χ^2^ Kruskal–Wallis test, * *p* < 0.05, ** *p* < 0.01.

**Table 9:** Multivariate linear regression for predictors of negative effects of COVID-19.

F (2.238) = 33.08, *p* < 0.001, R^2^ = 0.21		β +	SE ++	b ‡	*p*
Gender	Female				
	Male	−0.109	0.016	−0.383	<0.001
Multiple ACEs		0.025	0.005	0.278	<0.001

β + = standardized coefficient, SE ++ = standard error, b ‡ = unstandardized coefficient.

**Table 10:** Multivariate linear regression analysis for predictors of trait anxiety.

F (3.237) = 54.38, *p* < 0.001, R^2^ = 0.40		β +	SE ++	b ‡	*p*
Gender	Female				
	Male	−0.036	0.010	−0.190	0.001
Number of ACEs		0.014	0.003	0.230	<0.001
Scale of negative effects of the pandemic		0.153	0.020	0.444	<0.001

β + = standardized coefficient, SE ++ = standard error, b ‡ = unstandardized coefficient.

**Table 11:** Multivariate linear regression analysis for predictors of state anxiety.

F (2.243) = 19.46, *p* < 0.001, R^2^ = 0.23		β +	SE ++	b ‡	*p*
Class	1st middle school				
	2nd middle school	0.031	0.012	0.185	0.008
	3rd middle school	0.023	0.012	0.128	0.062
Number of ACEs		0.011	0.003	0.206	0.001
Scale of Negative Effects of the Pandemic		0.110	0.018	0.358	<0.001

β + = standardized coefficient, SE ++ = standard error, b ‡ = unstandardized coefficient.

## Data Availability

The data that support the findings and all questionnaires of this study are available from the corresponding author upon reasonable request. The data are not publicly available due to restrictions (they contain information that could compromise the privacy of research participants). Data access may be obtained by submitting a request to the Zenodo Repository (https://doi.org/10.5281/zenodo.8289606, accessed on 11 July 2024).

## References

[1] Felitti V.J., Anda R.F., Nordenberg D., Williamson D.F., Spitz A.M., Edwards V., Koss M.P., Marks J.S. (1998). Relationship of childhood abuse and household dysfunction to many of the leading causes of death in adults: The Adverse Childhood Experiences (ACE) Study. Am. J. Prev. Med..

[2] Alhowaymel F., Kalmakis K., Jacelon C. (2021). Developing the Concept of Adverse Childhood Experiences: A Global Perspective. J. Pediatr. Nurs..

[3] Struck S., Stewart-Tufescu A., Asmundson A.J.N., Asmundson G.G.J., Afifi T.O. (2021). Adverse childhood experiences (ACEs) research: A bibliometric analysis of publication trends over the first 20 years. Child Abus. Negl..

[4] Elmore A.L., Crouch E. (2020). The Association of Adverse Childhood Experiences with Anxiety and Depression for Children and Youth, 8 to 17 Years of Age. Acad. Pediatr..

[5] Finkelhor D., Shattuck A., Turner H., Hamby S. (2015). A revised inventory of Adverse Childhood Experiences. Child Abus. Negl..

[6] Lew D., Xian H. (2019). Identifying Distinct Latent Classes of Adverse Childhood Experiences Among US Children and Their Relationship with Childhood Internalizing Disorders. Child Psychiatry Hum. Dev..

[7] Boullier M., Blair M. (2018). Adverse childhood experiences. Paediatr. Child Health.

[8] Gkintoni E., Kourkoutas E., Yotsidi V., Stavrou P.D., Prinianaki D. (2024). Clinical Efficacy of Psychotherapeutic Interventions for Post-Traumatic Stress Disorder in Children and Adolescents: A Systematic Review and Analysis. Children.

[9] Merrick M.T., Ford D.C., Ports K.A., Guinn A.S. (2018). Prevalence of Adverse Childhood Experiences From the 2011-2014 Behavioral Risk Factor Surveillance System in 23 States. JAMA Pediatr..

[10] Cabral M.D., Patel D.R., Kim Y.-K. (2020). Risk Factors and Prevention Strategies for Anxiety Disorders in Childhood and Adolescence. Anxiety Disorders: Rethinking and Understanding Recent Discoveries.

[11] Ghandour R.M., Jones J.R., Lebrun-Harris L.A., Minnaert J., Blumberg S.J., Fields J., Bethell C., Kogan M.D. (2018). The Design and Implementation of the 2016 National Survey of Children’s Health. Matern. Child Health J..

[12] Merikangas K.R., He J., Burstein M., Swendsen J., Avenevoli S., Case B., Georgiades K., Heaton L., Swanson S., Olfson M. (2011). Service utilization for lifetime mental disorders in U.S. adolescents: Results of the National Comorbidity Survey-Adolescent Supplement (NCS-A). J. Am. Acad. Child Adolesc. Psychiatry.

[13] Kim I., Galván A., Kim N. (2021). Independent and cumulative impacts of adverse childhood experiences on adolescent subgroups of anxiety and depression. Child. Youth Serv. Rev..

[14] Kovács-Tóth B., Oláh B., Papp G., Szabó I.K. (2021). Assessing adverse childhood experiences, social, emotional, and behavioral symptoms, and subjective health complaints among Hungarian adolescents. Child Adolesc. Psychiatry Ment. Health.

[15] Farmakopoulou I. (2022). From “In the House Not in the City” to “In the House in the City” in Domestic and Gender-Based Violence. Child Adolesc. Psychiatry.

[16] Farmakopoulou I., Liakopoulou M., Hadzara V., Kolaitis G. (2012). Social Care or Lost Atlantis? Children’s Journey from Removal from their Family Environment to their Final Rehabilitation; Reflections and Social Policy Proposals. Soc. Work..

[17] Crouch E., Probst J.C., Radcliff E., Bennett K.J., McKinney S.H. (2019). Prevalence of adverse childhood experiences (ACEs) among US children. Child Abus. Negl..

[18] Chi X., Jiang W., Guo T., Hall D.L., Luberto C.M., Zou L. (2022). Relationship between adverse childhood experiences and anxiety symptoms among Chinese adolescents: The role of self-compassion and social support. Curr. Psychol..

[19] Lee H.Y., Kim I., Nam S., Jeong J. (2020). Adverse childhood experiences and the associations with depression and anxiety in adolescents. Child. Youth Serv. Rev..

[20] Bera L., Souchon M., Ladsous A., Colin V., Lopez-Castroman J. (2022). Emotional and Behavioral Impact of the COVID-19 Epidemic in Adolescents. Curr. Psychiatry Rep..

[21] Buzzi C., Tucci M., Ciprandi R., Brambilla I., Caimmi S., Ciprandi G., Marseglia G.L. (2020). The psycho-social effects of COVID-19 on Italian adolescents’ attitudes and behaviors. Ital. J. Pediatr..

[22] de Figueiredo C.S., Sandre P.C., Portugal LC L., Mázala-de-Oliveira T., da Silva Chagas L., Raony Í., Ferreira E.S., Giestal-de-Araujo E., Dos Santos A.A., Bomfim P.O.-S. (2021). COVID-19 pandemic impact on children and adolescents’ mental health: Biological, environmental, and social factors. Prog. Neuro-Psychopharmacol. Biol. Psychiatry.

[23] Ma L., Mazidi M., Li K., Li Y., Chen S., Kirwan R., Zhou H., Yan N., Rahman A., Wang W. (2021). Prevalence of mental health problems among children and adolescents during the COVID-19 pandemic: A systematic review and meta-analysis. J. Affect. Disord..

[24] Racine N., McArthur B.A., Cooke J.E., Eirich R., Zhu J., Madigan S. (2021). Global Prevalence of Depressive and Anxiety Symptoms in Children and Adolescents During COVID-19: A Meta-analysis. JAMA Pediatr..

[25] Gkintoni E., Koutsopoulou I., Antonopoulou H., Christopoulos P. Consequences of the COVID-19 Pandemic on Greek Students’ Mental Health: Quality of Life and Trauma Stressful Events Correlation. Proceedings of the ICERI Proceedings 2021.

[26] Gkintoni E., Ortiz P.S. (2023). Neuropsychology of Generalized Anxiety Disorder in Clinical Setting: A Systematic Evaluation. Healthcare.

[27] Zhou S.-J., Zhang L.-G., Wang L.-L., Guo Z.-C., Wang J.-Q., Chen J.-C., Liu M., Chen X., Chen J.-X. (2020). Prevalence and socio-demographic correlates of psychological health problems in Chinese adolescents during the outbreak of COVID-19. Eur. Child Adolesc. Psychiatry.

[28] Lackova Rebicova M., DankulincovaVeselska Z., Husarova D., MadarasovaGeckova A., van Dijk J.P., Reijneveld S.A. (2019). The Number of Adverse Childhood Experiences Is Associated with Emotional and Behavioral Problems among Adolescents. Int. J. Environ. Res. Public Health.

[29] Raymond C., Provencher J., Bilodeau-Houle A., Leclerc J., Marin M.-F. (2022). A longitudinal investigation of psychological distress in children during COVID-19: The role of socio-emotional vulnerability. Eur. J. Psychotraumatol..

[30] Kaufman J., Birmaher B., Brent D., Rao UM A., Flynn C., Moreci P., Ryan N. (1997). Schedule for affective disorders and schizophrenia for school-age children-present and lifetime version (K-SADS-PL): Initial reliability and validity data. J. Am. Acad. Child Adolesc. Psychiatry.

[31] Kolaitis G., Korpa T., Kolvin I., Tsiantis J. (2003). Schedule for affective disorders and schizophrenia for school-age children-present episode (K-SADS-P): A pilot inter-rater reliability study for Greek children and adolescents. Eur. Psychiatry.

[32] Spielberger C.D., Edwards C.D. (1973). STAIC Preliminary Manual for the State-Trait Anxiety Inventory for Children (‘How I Feel Questionnaire’).

[33] Psychountaki M., Zervas Y., Karteroliotis K., Spielberger C. (2003). Validity and reliability of the State-Trait Anxiety Inventory for children in Greek population. Eur. J. Psychol. Assess..

[34] Thorndike R.M. (1995). Book Review: Psychometric Theory (3rd ed.) by Jum Nunnally and Ira Bernstein New York: McGraw-Hill, 1994, xxiv + 752 pp. Appl. Psychol. Meas..

[35] Gkintoni E., Skokou M., Gourzis P. (2024). Integrating Clinical Neuropsychology and Psychotic Spectrum Disorders: A Systematic Analysis of Cognitive Dynamics, Interventions, and Underlying Mechanisms. Medicina.

[36] Magklara K., Lazaratou H., Barbouni A., Poulas K., Farsalinos K. (2022). The impact of COVID-19 lockdown on children’s and adolescents’ mental health in Greece. Child. Soc..

[37] Kolaitis G., Olff M. (2017). Psychotraumatology in Greece. Eur. J. Psychotraumatol..

[38] Koutsopoulou I., Grace E., Gkintoni E., Olff M. (2024). Validation of the Global Psychotrauma Screen for adolescents in Greece. Eur. J. Trauma Dissociation.

[39] Scully C., McLaughlin J., Fitzgerald A. (2020). The relationship between adverse childhood experiences, family functioning, and mental health problems among children and adolescents: A systematic review. J. Fam. Ther..

[40] Lacey R.E., Minnis H. (2020). Practitioner Review: Twenty years of research with adverse childhood experience scores—Advantages, disadvantages and applications to practice. J. Child Psychol. Psychiatry.

[41] McLaughlin K.A., Greif Green J., Gruber M.J., Sampson N.A., Zaslavsky A.M., Kessler R.C. (2012). Childhood adversities and first onset of psychiatric disorders in a national sample of US adolescents. Arch. Gen. Psychiatry.

[42] Gkintoni E. (2023). Clinical neuropsychological characteristics of bipolar disorder, with a focus on cognitive and linguistic pattern: A conceptual analysis. F1000Research.

[43] Farmakopoulou I., Lekka M., Gkintoni E. (2024). Clinical Symptomatology of Anxiety and Family Function in Adolescents—The Self-Esteem Mediator. Children.

[44] Sortwell A., Evgenia G., Zagarella S., Granacher U., Forte P., Ferraz R., Ramirez-Campillo R., Carter-Thuillier B., Konukman F., Nouri A. (2023). Making neuroscience a priority in Initial Teacher Education curricula: A call for bridging the gap between research and future practices in the classroom. Neurosci. Res. Notes.

[45] D’Costa S., Rodriguez A., Grant S., Hernandez M., Alvarez Bautista J., Houchin Q., Brown A., Calcagno A. (2021). Outcomes of COVID-19 on Latinx youth: Considering the role of adverse childhood events and resilience. Sch. Psychol..

[46] Chen F., Zheng D., Liu J., Gong Y., Guan Z., Lou D. (2020). Depression and anxiety among adolescents during COVID-19: A cross-sectional study. Brain Behav. Immun..

[47] de Araújo L.A., Veloso C.F., Souza M.d.C., de Azevedo J.M.C., Tarro G. (2021). The potential impact of the COVID-19 pandemic on child growth and development: A systematic review. J. Pediatr..

[48] van Oort F., Greaves-Lord K., Verhulst F.C., Ormel J., Huizink A. (2009). The developmental course of anxiety symptoms during adolescence: The TRAILS study. J. Child Psychol. Psychiatry Allied Discip..

[49] Van der Kolk B.A. (2017). Developmental Trauma Disorder: Toward a rational diagnosis for children with complex trauma histories. Psychiatr. Ann..

[50] Ghazali S.R., Elklit A., Balang R.V., Sultan M.A., Kana K. (2014). Preliminary findings on lifetime trauma prevalence and PTSD symptoms among adolescents in Sarawak Malaysia. Asian J. Psychiatry.

[51] Gkintoni E., Kourkoutas E., Vassilopoulos S.P., Mousi M. (2024). Clinical Intervention Strategies and Family Dynamics in Adolescent Eating Disorders: A Scoping Review for Enhancing Early Detection and Outcomes. J. Clin. Med..

[52] Hovenkamp-Hermelink JH M., Jeronimus B.F., Myroniuk S., Riese H., Schoevers R.A. (2021). Predictors of persistence of anxiety disorders across the lifespan: A systematic review. Lancet Psychiatry.

[53] McKay M.T., Cannon M., Chambers D., Conroy R.M., Coughlan H., Dodd P., Healy C., O’Donnell L., Clarke M.C. (2021). Childhood trauma and adult mental disorder: A systematic review and meta-analysis of longitudinal cohort studies. Acta Psychiatr. Scand..

[54] Solmi M., Radua J., Olivola M., Croce E., Soardo L., Salazar de Pablo G., Il Shin J., Kirkbride J.B., Jones P., Kim J.H. (2022). Age at onset of mental disorders worldwide: Large-scale meta-analysis of 192 epidemiological studies. Mol. Psychiatry.

[55] Danese A. (2020). Annual Research Review: Rethinking childhood trauma-new research directions for measurement, study design and analytical strategies. J. Child Psychol. Psychiatry.

[56] Lewis S.J., Arseneault L., Caspi A., Fisher H.L., Matthews T., Moffitt T.E., Odgers C.L., Stahl D., Teng J.Y., Danese A. (2019). The epidemiology of trauma and post-traumatic stress disorder in a representative cohort of young people in England and Wales. Lancet. Psychiatry.

[57] Liming K.W. (2018). Examining the Differing Effects of Economic Hardship and Poor Maternal Wellbeing on Cumulative Exposure to Adverse Childhood Experiences. J. Child Adolesc. Trauma.

[58] Stavridou A., Stergiopoulou A.-A., Panagouli E., Mesiris G., Thirios A., Mougiakos T., Troupis T., Psaltopoulou T., Tsolia M., Sergentanis T.-N. (2020). Psychosocial consequences of COVID-19 in children, adolescents and young adults: A systematic review. Psychiatry Clin. Neurosci..

[59] Beesdo K., Knappe S., Pine D.S. (2009). Anxiety and anxiety disorders in children and adolescents: Developmental issues and implications for DSM-V. Psychiatr. Clin. N. Am..

[60] Merikangas K.R., He J.P., Burstein M., Swanson S.A., Avenevoli S., Cui L., Swendsen J. (2010). Lifetime prevalence of mental disorders in US adolescents: Results from the National Comorbidity Survey Replication–Adolescent Supplement (NCS-A). J. Am. Acad. Child Adolesc. Psychiatry.

